# Hamilton Labbat on the Use and Abuse of Restraint in the Treatment of the Insane

**Published:** 1849-04-01

**Authors:** 


ART. III.—*An Essay on the Use and Abuse of Restraint in the Management of the Insane, including some Remarks on the Origin and Nature of their Disease; with copious Notes.* By HAMILTON LABATT, A.B., T.C.D., Fellow of the Royal College of Surgeons of Ireland, &c. Hodges and Smith. Dublin. pp. 76.

AMONGST physicians and philanthropists, no subject connected with insanity has deservedly occupied so much attention as the important inquiry respecting the use and abuse of mechanical restraint in the treatment of the insane.

We are delighted that this most interesting question has been so fully—nay, enthusiastically, discussed; and although some may perhaps think that exaggeration, according to their view of the controversy, has occasionally characterized the arguments advanced by the advocates for the total abolition of all kinds of mechanical restraint, in every form of insanity, still, the injurious consequences resulting from its indiscriminate employment are so very evident, obvious, and palpable, even to a casual observer, that we think there cannot be any doubt entertained respecting the fundamental principle—*that the system of non-restraint ought to be the established rule in all asylums for lunatics: whilst the exceptions to such an axiom should be indeed of very, very rare occurrence.*

Before entering specially on an examination of the publication now before us, it may be interesting to cast a retrospective glance upon the defective condition and management of some of the asylums in England and elsewhere, during the olden, if not more recent, times; in order to contrast their present improved state with the horrifying accounts contained in authentic documents, which describe occurrences unfortunately then too common; and that no longer back than the commencement of the present century. It will be also instructive to advert briefly to the barbarous treatment the insane are still subjected to in many parts of the world, where the doctrines of non-restraint, and the modern more humane method of treating the insane, have unfortunately not yet been adopted. Even in France, so much psychologically in advance of other countries, we are told by writers of the day, that it was formerly not uncommon for French physicians to travel to England in order to gain information on this important subject, so as to improve the system formerly pursued in their own badly-regulated establishments. Such were the motives that induced the benevolent Tenon, towards the end of the last century, to visit London, expressly to see the provincial and two metropolitan hospitals for the insane. This excellent physician was one of the first individuals who, in France, proposed to remove all the lunatic patients, then confined in the ill-ventilated and unhealthy wards of the Hotel Dieu, of Paris, to an asylum specially appropriated for their reception; which soon led to the foundation of those noble institutions for the insane, where Pinel, Esquirol, and other physicians well known to fame, have laboured in the cause of science and humanity.

In Italy, Germany, and in Holland, the march of improvement has likewise proceeded, as every person can testify, who has visited, within these few years past, the asylum at Aversa, near Naples; Sonnenstein, in Saxony; Siegburg, in Rhenish Prussia; and the madhouse of Utrecht: even at Vienna, in which capital, many years ago, we witnessed in the old and circular asylum, sad scenes and modes of treating the insane inmates which are now no longer pursued. In some of the Italian asylums, especially at Genoa, we also saw many of the patients chained by the hands and feet. Fortunately, matters are now much improved, as well in Italy as Germany; and at Vienna, a new hospital for lunatics is being constructed upon improved principles, in order to correct the defects of the ancient and badly-arranged building; as also to carry out the more humane method of treating the insane, at present adopted by the psychological physicians of England and France. Again, throughout North America, much has likewise been accomplished in the same benevolent cause, as shown by the many recent valuable reports emanating from various asylums of that country. But in other parts of the world, and even in places where Christians rule supreme, the condition of the poor maniac is still sometimes most deplorable, and often very little removed from barbarism; thus proving, that the modern doctrines of non-restraint, and improved moral treatment of mental maladies, have not yet penetrated into these benighted regions, still less into countries like Spain, Egypt and Turkey.

That such condemnatory asseverations are not made without proof of their existence, it is only necessary to make one or two references to recent observers, who, from personal knowledge, obtained during their travels, describe the treatment to which afflicted lunatics are actually subjected in these countries, and the management of the asylums wherein they are confined. For example, Mr. Ford, in his recent learned and remarkable publication,* states, that the lunatic asylum at Toledo is no honour to Spain. There is no attempt at classification. The inmates are usually crowded together in one confusion of dirt and misery, where they howl at each other, chained like wild beasts, and treated even worse than criminals; for the passions of the most furious are augmented by the savage lash. There is not even a curtain to conceal the sad necessities of these human beings, now reduced to animals; everything is public, even unto death, whose last groan is mingled with the frantic laugh of the surviving spectators. In some rare cases, the maniac is confined in solitary cells. Of these, many, when first sent, were *not mad,* but put out of the way by friends and relatives. This establishment is a shame to Toledo; and in 1843, the keepers always conducted any visitor to the cage, or den, in which the wife of a celebrated Captain-General of Catalonia was shown, as a special object of cruel curiosity, and made a public *show,* although permitted to wallow in naked filth. Again, in the lunatic asylum at Granada—the oldest establishment, we believe, of the kind in Europe, having been founded by Ferdinand and Isabella—matters are by no means in a better condition, its filth and mismanagement, according to the authority of Mr. Ford, being scandalous.

In the New World, or South America, the asylums are quite as bad as in Spain; and we are informed by Von Tschudi, when describing his recent visit to Lima, that the lunatic hospital of St. André in that capital is opened on every 30th November, on St. Andrew's day, for the admission of the public into the wards occupied

* "The Handbook of Spain."

by the insane patients. On that occasion, one of the favourite amusements of the inhabitants of Lima, fashionable as otherwise, is to go to St. André's Hospital, to see the lunatics. It is melancholy to observe, according to Von Tschudi's narrative, these unfortunate beings thus made the object of public exhibition, and irritated by the idle throng who go to stare at them. The collection of alms from the numerous visitors, who come to see this revolting show, was doubtless the motive for keeping up so exceedingly reprehensible a custom. This reminds us of a similar and equally injurious practice, which prevailed almost daily in a London madhouse till towards the end of the last century; when, according to an account of Bethlem Hospital, published in 1783, by the Rev. Thomas Bowen, the chaplain to the institution, a revenue of at least 400*l.* per annum was obtained from the unlimited admission of visitants to the hospital, whom very often an idle and wanton curiosity drew to that region of distress; and as each person paid about one penny, according to the finding of the committee of the 12th March, 1742, about ninety thousand visitors were sometimes admitted in one year. The crowd was often so great, that to prevent disturbances, the porter was annually made a constable, and attended with the other servants to keep order. However, this abuse became so general, and the injury inflicted upon the patients was so apparent, that in 1770 all indiscriminate permission to visit the hospital was very properly forbidden. The same improper licence of visiting the inmates was also formerly permitted at many continental asylums for the insane; where the lunatics were confined in cages, through the bars of which food and straw were thrust in, as if to wild beasts. In this condition, these unfortunate human beings were publicly exhibited to visitors, who paid a certain sum to see them, as at a menagerie. Happily such barbarous proceedings are not now to be witnessed, we believe, in any part of civilized Europe, and even in those dark barbarian regions where such injurious customs still prevail, they must inevitably give way before the sure but certain advance of knowledge and civilization.

Amongst Mahomedans and Turks the treatment adopted towards lunatics, and the cruelties they are often subjected to, is even much worse than in those Christian countries to which allusion has just been made. For instance, at Grand Cairo, when Von Orlich visited, only the other day, the lunatic hospital of the Egyptian capital, he found, as related in his "Travels to India," that a narrow yard, surrounded by lofty buildings, the lower rooms of which contained small cells or cages, like those for wild beasts, contained at the time of his visit between twenty and thirty inmates, most of whom were in chains, almost naked, and covered with filth. The keeper told Von Orlich that the method of cure usually adopted towards these poor creatures was scanty food, a shower-bath three times a day, and corporal punishment! No description could be more graphic, however harrowing it proves to the feelings of a humane spectator when visiting such a receptacle, whilst it truly indicates the deplorable condition to which these poor victims of insanity have been reduced in this country, although bordering on Syria, where Turks also rule, and in which lunatics appear even favoured beings, being there known by the name of Santons.

Again, at Constantinople, according to Dr. John Davy, who resided some time in that metropolis, the management of lunatic asylums, and the treatment of the inmates, seem equally as deplorable as in Egypt. In the madhouse of the Turkish capital, the lunatics are kept in cold cells in the winter, although snow is on the ground, with unglazed windows. The poor men are chained by the neck to the wall, with a heavy iron chain six feet in length, being the space to which their exercise is limited. No medical aid is afforded them. The inmates are open to the public gaze, and subjected to irritation of an aggravated kind, from mischievous boys and lads, who torment, and even strike the violent and furious. The asylum is near a menagerie, and the visitor must pass through the yard containing cages, in which a few wild beasts are exhibited, in order to enter that containing the cells of the lunatics; and payment for both exhibitions is the same. Miss Pardoe, in her recent "Tour to Constantinople," fully confirms the account given by Dr. Davy; indeed, other authors have published similar statements; whilst it has been remarked, that the most painful object, connected with the scenes witnessed in this den of iniquity, was the heavy chain and collar of iron worn by each of the lunatics confined.

The disclosures revealed by the parliamentary investigations at the early part of the current century, show that, in this country, the management of asylums was very defective, and the treatment of the lunatics immured therein most objectionable; whilst transactions then took place, in some English insane establishments, almost as bad as the atrocities just described to be still common in Spain and Turkey. In France, Pinel fortunately commenced his philanthropic crusade against the antiquated system pursued towards insane patients, before the period now alluded to; and although Samuel Tuke had also laboured much in aid of the same benevolent object, even so early as 1792, it is only of late years that the broad principle of doing away with all mechanical restraint whatever in the management of lunatics has been laid down and acted upon by those English physicians who, along with other philanthropists, now take the lead in this humane movement.

Among the various publications which have recently appeared bearing upon so interesting a subject as the treatment of lunatics without restraint, we have selected the one whose title is placed at the head of this article. The volume, although of small dimensions, we have chosen, from the circumstance of its being an advocate of the doctrine of non-restraint, and one which deserves perusal. If allowed to perpetrate a pun, without giving offence, where none is really intended, our feeling being quite otherwise, the book now under review may well be designated "LaBatt-*u* amongst the coercionists." Speaking metaphorically, in the language of sportsmen, the game is plentiful, and the result satisfactory; whilst looking on as by-standers or critics, we do say the feats performed in the good cause are creditable, and merit approbation. Preliminary to entering upon an examination of some of the important points discussed in the volume before us, we would take the liberty of remarking, that to our comprehension at least, some obscurity envelopes the circumstance alluded to in the author's preface, the whole merits of which we do not so fully understand as to venture upon giving any judicial opinion. We therefore afford the writer an opportunity of stating the case and arguments in his own words:—

"The cause, then, for the publication of this Essay, is at once declared. Under other circumstances, it might probably have rested in that oblivion, to which the selected manuscript has been hitherto unfortunately consigned. And much, indeed, is it to be regretted, that the object originally contemplated by the learned founder of the Prize should be frustrated by the non-appearance of that Essay—a production which, if published, would have afforded an opportunity for canvassing arguments designed to uphold physical restraint as a remedial agent in insane cases. And, on this account, the author entertains peculiar reasons for anxiety, inasmuch as opinions directly at variance with the views put forward in that manuscript are advocated in the following pages."

The allusion made in the above quotation would seem to imply that some irregularity had been committed in the adjudication of a prize requiring an explanation. This appears the more necessary, if it be wished that persons at a distance should understand all the *pros* and *cons* of the cause which is thus submitted to their judgment by way of appeal. If otherwise intended, it would be much better, in our humble opinion, to let the matter now rest in oblivion, especially seeing some time has elapsed since the occurrence took place; the more so, as the Irish College of Surgeons, according to the author, "well may congratulate itself on the significant and vigorous manifestation of spirit and determination which, to the honour and credit of our body be it stated, has been evinced to discountenance such means of superseding fair and unimpeachable competition." This "fiat" of so high a court seems conclusive.

Notwithstanding the reader may fairly entertain a feeling of incredulity and amazement, when perusing the details, recorded on indubitable authority, of the mode in which many unfortunate victims of mental alienation were formerly treated in some asylums, there cannot exist any doubt whatever, as the author states at the commencement of his Essay, that "to the system of cruel and unmitigated restraint, recorded as part of the treatment formerly adopted in lunatic asylums, we may attribute that reaction in public opinion which has happily terminated in the very rapid and almost universal revolution in the management of the insane."

Unquestionably, the many instances reported in books of the afflicted maniac's sufferings, his madness exasperated, and his disease confirmed by the lash or chain, are by far too well authenticated now to require any further proof or illustration; whilst learned professors, able physicians, and even individuals, otherwise humane, formerly sanctioned—nay, did not hesitate to inculcate upon their pupils—measures which would be now considered, and very justly, as harsh and cruel treatment. Thus Dr. Cullen recommended the medical practitioner to endeavour to produce a constant impression of fear upon the insane patient, and to inspire awe and dread in his weakened mind, in order to cure the existing mental disease. In fact, as was well said by De la Rive, of Geneva, when speaking of the great evils formerly existing, and the improper practices then prevalent in some English asylums for the insane, "one would think madmen were employed to torture madmen."

No impartial person can well deny but much reprehensible treatment, at one time, took place in many receptacles for the insane; nevertheless, the great majority of institutions for the reception and cure of lunatics, more particularly in the metropolitan districts, are now of a very different character to the description handed down to us in reports of similar places, during former days of darkness, when chains, belts, and straw were but too frequent appliances to control excited patients, and to relieve lazy attendants. A complete revolution has fortunately taken place, thanks to an enlightened public opinion, backed by philanthropy and medical science. Such being the fact, and as no good would supervene now to reproduce to our readers the details of cruelties, abuses, and frauds which were many years ago perpetrated in madhouses, according to the authority of Mr. J. S. Rogers, a surgeon in London, and to whose statements the author makes frequent reference, we think it will be much better to pass them over in silence, believing confidently that proceedings of a similar kind can very seldom or never again occur. The facts recorded by Mr. Rogers then belong to history, and attracted, certainly, much attention at the time they were brought forward; but as it could serve no beneficial purpose at present to recal the particulars to recollection, they well may be consigned to forgetfulness.

The first impetus in this country towards an improved system of treating lunatics was undoubtedly given by Mr. Tuke, about the end of the year 1793, as already stated, when a piece of ground, near York, was purchased by the Society of Friends, of which body he was a distinguished ornament. In the above-named locality, the now justly-celebrated Retreat was erected as an asylum for the insane. During the same year, but subsequent to the proceedings just mentioned, Pinel commenced his useful and never-to-be-forgotten labours in aid of the unfortunate maniacs confined at Bicêtre. Having first obtained the sanction of the revolutionary government, then dominant in France, Pinel immediately began those important reformatory measures in the treatment of lunatics which have since immortalized his name. As we cannot better illustrate this, the culminating point in Pinel's brilliant career, or place the matter more clearly or instructively before our professional brethren, we transcribe the statement made by the author in the subjoined paragraph:—

"There were about fifty whom he considered might, without danger to the others, be unchained; and he began by releasing twelve, with the sole precaution of having previously prepared the same number of strong waistcoats, with long sleeves, which could be tied behind the back if necessary. The first man on whom the experiment was to be tried was an English captain, whose history no one knew, as he had been in chains forty years. He was thought to be one of the most furious among them. His keepers approached him with caution, as he had, in a fit of fury, killed one of them on the spot with a blow of his manacles. He was chained more rigorously than any of the others. Pinel entered his cell unattended, and calmly said to him, 'Captain, I will order your chains to be taken off, and give you liberty to walk in the court, if you will promise me to behave well and injure no one.' 'Yes, I promise you,' said the maniac; 'but you are laughing at me; you are all too much afraid of me.' 'I have six men,' answered Pinel, 'ready to enforce my commands, if necessary. Believe me, then, on my word, I will give you your liberty if you will put on this waistcoat.' He submitted to this willingly, without a word. His chains were removed, and the keepers retired, leaving the door of the cell open. He raised himself many times from his seat, but fell again on it, for he had been in a sitting posture so long, that he had lost the use of his limbs. In a quarter of an hour, he succeeded in maintaining his balance, and with tottering steps came to the door of his dark cell. His first look was at the sky, and he cried out, enthusiastically, 'How beautiful!' During the rest of the day he was constantly in motion, walking up and down the staircases, and uttering short exclamations of delight. In the evening he returned, of his own accord, to his cell, where a better bed than he had been accustomed to had been prepared for him, and he slept tranquilly. During the two succeeding years which he spent in the Bicêtre, he had no return of his previous paroxysms, but even rendered himself useful by exercising a kind of authority over the insane patients, whom he ruled in his own fashion."

Although frequently quoted, indeed, scarcely a book on insanity is now published without some reference being made to this interesting proceeding on the part of Pinel, we nevertheless consider the facts cannot be too frequently repeated, or their importance too much exaggerated. For ourselves, we always feel gratified in the highest degree, when reading this most instructive illustration of the beneficial consequences of the humane treatment of lunatics, then so different from all previous methods. This interesting account of Pinel's proceedings at Bicêtre possesses besides, to our mind, especial value, from the circumstance that it represents the benevolent Frenchman kindly treating an unfortunate Englishman, with a degree of confidence to which he had, hitherto, been long a stranger; owing, doubtless, to the erroneous notions till then prevalent respecting the proper treatment of lunatics. It also gives, at the same time, an instructive and moral lesson to all mankind. In this country, the example of Pinel was zealously followed up, and the application of his principles has even been carried further than by many of the successors of that able physician on the other side of the English Channel. This we can assert from personal observation; for however much may have been otherwise accomplished in France, towards improving the condition of the victims of insanity, by amusements, occupation, and other auxiliaries, both moral and medical, personal restraint is certainly much more frequently employed by French, than by the generality of modern English practitioners. Besides, truth compels us to state distinctly, that even in some Parisian and provincial asylums for the insane we ourselves visited not long ago, the strait-waistcoat, or *camisole*, is by no means an uncommon appliance, especially to females labouring under excitement. This we witnessed in several instances, and the practice was even defended on the grounds of humanity, besides being considered beneficial to the patient.

Anxious to give the author an opportunity of expressing his sentiments respecting the question under discussion, we make the following quotation to show the object he wishes to illustrate by his treatise. How far the writer proves the position he has taken up, the reader will afterwards be able to judge.

"Notwithstanding the great weight of authority opposed to us, we have long been of opinion that it has been productive of the worst consequences; that under it every influence of a moral tendency is endangered, if not destroyed; and that its adoption has led, in many cases, to a forfeiture of that confidence so essentially necessary in our treatment; and which, under different circumstances, the patient would have reposed in his attendant. Such is the position we would maintain, and we entertain a sanguine hope of being able to prove, assisted in no slight degree by the statements of even the adopters of restraint, that whether we view its effects morally or physically, its use ought to be completely excluded from the precincts of a lunatic asylum."

Subsequently, a case is quoted in which a middle-aged man, labouring under great maniacal excitement, was placed under bodily restraint. While in this state, the medical officer in attendance removed the restraint, when the patient gradually became calmer, shook hands with the author, saying, "He would fight for him against any man," yet continued most indignant towards those who had imposed the coercion. Since we are not told whether the maniac was permanently benefited, or ultimately cured, the history of the case is therefore imperfect; although it is certainly an illustration, as stated by the author, of the necessity of gaining an ascendancy in the confidence of the patient, and of sympathizing with him in his feelings.

The author next proceeds to the consideration of the first measure of restraint which is usually adopted, preparatory to remedial treatment of the insane—namely, the separation of the patient from friends and relatives, by removal to a suitable place of residence, where new trains of ideas will arise, and former associations may be removed from the mind as much as possible. On this point the author agrees with most other physicians who have investigated the subject; and we coincide with him, as also other authorities, in thinking that the presence of strangers often suspends the dilirium of insane patients, either by the influence of new impressions, or from a secret feeling of self-respect, which induces even lunatics to exert some control over themselves. For it has been remarked that individuals have, in the midst of the most violent maniacal excitement, suddenly become tranquil on the appearance of their physician, accompanied by a stranger. Respecting this point in the treatment of the insane, the author remarks—

"The separation, to be effectual, must be *absolute* and *early* in its adoption. *Absolute*, because even a single visit at an unseasonable period, is calculated to renew associations and ideas in which, probably, the disease originated; and, consequently, may be the cause of as much evil as one conducted at the proper and fitting period, and with discretion, may be productive of advantage. Of this we have the strongest evidence adduced by Willis and Esquirol; the former states that in England he found the treatment of foreigners, who had so few opportunities of seeing their friends, was much more successful than that of the English; and the latter authority experienced greater success amongst strangers coming to Paris for treatment. Indeed, M. Georget objects to private asylums on the distinct grounds that the patient is more likely to see his friends in such institutions. Besides, we are to recollect that there is no class of persons more likely to be betrayed into injudicious observations than those so deeply interested as relatives must naturally be; and it is quite impossible they can possess that address which we shall hereafter see is of so much moment, and so difficult to be acquired."

Indubitably, the removal of insane patients from former scenes and localities, when their mental malady first makes its appearance, is often of the greatest benefit; and also, the sooner they are placed under proper treatment and surveillance, the more likely will their recovery be accomplished. This opinion, entertained by the author, is fully borne out by experience. Dr. Willis, for instance, declares that nine out of ten of the lunatics who were placed under his care recovered during the first three months. Dr. Finch reports the proportion, in his experience, amounted to sixty-one in every sixty-nine; whilst, at the York Retreat, according to Mr. Tuke, the ratio of recoveries was seven out of every eight patients admitted. Indeed, the advantages of early treatment, in all cases of mental disease, is so well established, that every day's delay, after the first manifestations of insanity, is not only so much valuable time lost, but the evil consequences thereby occasioned become also greatly augmented. The importance of this truth cannot be too strongly impressed upon the minds of friends or relatives; and however painful it may seem to their own feelings, the idea of sending an afflicted relation to a public or private asylum, there to remain under the care of strangers, the proceeding is real kindness to all parties interested, whilst it gives a much greater chance of recovery. Of course, the institution to which the insane patient is consigned, should be a well managed asylum, and under the superintendence of trustworthy and experienced individuals.

Subsequently, the author discusses the pathology of mental diseases; upon which point he acknowledges considerable difference of opinion is still prevalent amongst medical men; but he nevertheless hopes that future investigations will clear up the present difficulty. In our opinion, this important question is not so unsettled as the author would seem to imply; and we might appeal to many recent dissections, made both by continental and English pathologists, illustrative of this most interesting branch of medical science. No necessity, therefore, exists for attempting to gloss it over by any ingenious or plausible theory. However, let the writer speak for himself:—

"According to Franck, Guislain, and Nasse of Bonn, with others, insanity is essentially a disease of the organic structure of the body; whilst, on the other hand, there are many (chiefly German authorities) who assert that madness is essentially a disease of the mind, and at the same time they adduce cases where no organic disease could be found. But, recollecting the utter impossibility of appreciating the first trace of organic disease, as also the very minute alteration in structure which may produce most serious symptoms, we ought certainly to be slow in acceding to such views. Professor Heinroth declares it to be *a moral depravity;* this theory we have quite sufficient arguments to controvert."

But even amongst those physicians who consider insanity to be a disease of organic structure, there prevails also, according to the author, a variety of opinions:—

"Some—as Georget, Foville, Cox, Cullen, and Haslam—assert that it is an idiopathic affection of the brain, and that the diseases found in other parts of the body are merely accidental, and consequent thereon. Others regard the primary disease to be in the stomach, as Broussais and his followers, who affirm that it always consists in irritation of the trisplanchnic apparatus. Pinel describes it as spreading from that organ as a centre. Lastly, we may refer to the views, in some respects peculiar, of Maximilian Jacobi, as first brought forward in his 'Collections for the Treatment of Disorders of the Mind,' (Sammlungen für die Heilkunde der Gemüthskrankheiten,) and more fully enlarged on in his subsequent works. This distinguished author, altogether opposed to the doctrine that would refer insanity to causes purely mental or moral, asserts that in every instance it is the consequence of functional or organic disease in some part of the system."

After several additional observations, being unwilling, unaided by visible proofs, to argue on a bare gratuitous assumption of its positive presence, the writer acknowledges—

"For our own part, in the absence of all theoretical bias, we cannot adhere exclusively to any of the views proposed respecting the cause of the disease in question. A very limited experience teaches us the influence of various organic changes over the operations of the intellectual principle; whereas, on the other hand, it is equally certain that cases of insanity do occur, where no lesion of internal organs can be discovered, and the disordered mind is presented as the sole and prominent malady."

The author, in a subsequent page, also remarks—"Whatever may be the primary seat of the disease, or whatever its cause, be it physical or moral, we are fully convinced as to the pernicious tendency of bodily restraint in the treatment of the insane." We fully coincide in such opinions, although the author does not speak so decidedly in another paragraph, when he says, in allusion to the practice pursued at the Hanwell Asylum—"We unhesitatingly affirm, there would be more humanity in having recourse to even an unqualified system of restraint, than to attempt, with insufficient means, what might only lead to scenes of suicide and bloodshed." We do not altogether understand the meaning intended to be conveyed by the above remark in a work whose express object is apparently to point out to the profession the evil consequences of employing personal restraint in the treatment of lunatics. We are by no means advocates of a dogmatic or authoritative style when discussing scientific subjects, especially disputed questions of pathology and medical practice; nevertheless, we like to hear the opinions of any writer, whose work it may be our lot to peruse, distinctly yet freely expressed; as likewise the principal facts detailed, and the chief arguments succinctly stated, upon which the conclusions promulgated are virtually founded. Now, with every respect for the learned writer, we confess, both in regard to the pathology of mental diseases and the employment of restraint in the treatment of insanity, there still remains, on our mind at least, after reading the volume before us, some degree of ambiguity respecting the exact opinions entertained by the author. That he is a conscientious opponent of personal restraint, we doubt not for a moment; but, in regard to the pathology of insanity, whether he is a vitalist or a decided follower of the anatomists, who consider mental alienation as always connected with diseased changes of structure in the brain and membranes, does not appear sufficiently explained, even after a very careful perusal, on our part, of the publication under review.

Before taking leave of the author, we would remark, with all respect for his literary labours, while we approve of the earnestness exhibited for the cause he advocates, in our opinion, the writer has not done full justice to himself, or the important subject discussed in his pages. Instead of giving copious notes illustrating the views of other authorities, a better method, and certainly more instructive, would have been, if the author had detailed, at greater length, the results of his personal experience, and particularly the accumulated facts which he must have collected from his own extensive observation. There is no deficiency whatever, either in the character or variety of the floating opinions prevalent amongst medical men, at the present time. Indeed, we have now rather an abundance of speculative doctrines in medical literature, especially since the many recent German importations. But in an utilitarian age like the nineteenth century, and still more so, in such a matter-of-fact country as England, which may truly be called the land of *cui bono philosophers*, the essential requisites which students and critics most value in books written to illustrate particular doctrines, are, above all, a strong array of data, statistical or otherwise, and verified by ample experience; so that correct conclusions may be thereby legitimately deduced. Opinions can be often controverted, and, besides, are sometimes erroneous; but well-established facts are most important, always useful, and can never be set aside by any reasoning, however plausible. In the treatise before us, there are undoubtedly many interesting statements, illustrated by data, both original, and derived from other sources; at the same time, that several of the conclusions enunciated by the author, are judicious, and fully borne out by the premises he has brought forward. Nevertheless, we candidly avow, instead of reading as a postscript, at the end of the publication now in our hands, the intimation that the writer "had omitted three statistical tables, which were in the original MS.," we should have been much more gratified by the perusal of these documents, before closing the volume just read, which we finally do with every good feeling towards the author, whilst acknowledging his zealous advocacy of the humane cause he has undertaken to defend before a jury of our professional brethren.

So much for the volume under review. Previous, however, to laying down our critical pen, we purpose, as stated in a former page, to make a few general observations respecting the recent progress and present position of the non-restraint doctrine in England. The subject is highly interesting, and, if space permitted, it would be easy to enlarge thereon at great length. Still, we must resist temptation, and therefore will confine our present notice within narrow limits—

the more so, as perhaps we may seem to have been already somewhat discursive.

In Great Britain, the non-restraint system of treating insane patients undoubtedly originated at the Lincoln Asylum; and to the authorities of that establishment, notwithstanding the difficulties they encountered at the commencement of their benevolent labours, belongs the honour of proclaiming, as well as of carrying into practice, that great principle; and they well deserve our thanks for their efforts in the cause of humanity. The example thus set at Lincoln was speedily followed in other lunatic institutions; and now, it may be justly said, the application and carrying out the doctrine of non-restraint has become so universal throughout the country, that in public, as also in private establishments for the reception of persons labouring under mental disease, the exceptions to this humane axiom are quite as rare as it was formerly uncommon to place confidence in lunatics, or treat them with kindness and consideration. Fortunately, the obsolete and erroneous notions, at one time prevalent respecting the mode of managing excited maniacs, alike injurious to the afflicted victims of insanity, as they were revolting to the best feelings of our nature, have all passed away, and given place to a much milder, and far more appropriate method of practice.

Amongst the leaders of this philanthropic movement, indeed the chief promoter and advocate, who has done so much for the holy cause, is indubitably Dr. Conolly; to whom belongs the credit, not certainly of inventing, or first applying the practice of non-restraint in the treatment of lunatics, but of reducing the measure to a practical system, and enforcing the principle on a large scale. To us who are spectators, chroniclers, and the critics of passing events having reference to the economy of lunatic asylums, and the treatment of the inmates therein confined, it is most gratifying to perceive that the eyes of the medical profession, and indeed of the whole civilized world, are now fully opened to the perfect practicability of treating lunatics upon the principles laid down by the enlightened advocates of non-restraint; whilst its great superiority over former systems—unfortunately characterised by harshness and physical confinement—seems so well established by ample experience, that it is every day gaining converts from former opponents, and will we hope soon have no enemy to contend against, at least in this country.

Considering it must be interesting to describe shortly the progress recently made in the path of improvement, in order to exhibit the marked diminution in the use of physical means of coercion, that has taken place in many lunatic asylums; we would refer, by way of illustration, to the reports published by several of these institutions, in which formerly restraint was frequently had recourse to during the treatment of insane patients. At the Lincoln Asylum, where the first impetus was given to the new views respecting the disuse of restraint, it appears that, in the year 1830, from forty to seventy patients were reported to be under restraint; the average number of inmates being about one hundred. Subsequently, more decided alterations in the system pursued at this institution were established, both in regard to the kind of instruments adopted, and the number of patients under restraint; until, at last, the total abolition of all physical means of coercion was announced, with the most beneficial consequences.

At the Retreat, near York, so early as 1813, when detailing the means of personal restraint then employed in the institution, Mr. Tuke observed, in his report, with regard to the necessity of coercion, he had no hesitation in saying, that it would diminish or increase, according as the moral treatment of the patient was more or less judicious. In 1841, although the officers of the Retreat had not hitherto thought it right, in every case, to dispense with the use of all mild and protecting means of personal restraint, it was very much diminished, notwithstanding, in some instances, they may have regarded its application as the least irritating, and therefore the kindest method of control. Two years afterwards, Dr. Thurnham, the experienced resident medical officer of the institution, stated, in one of his reports, that, in practice, personal restraint had, by degrees, been almost entirely abolished. Afterwards we are informed, that no instance of its application had occurred since January, 1843. These are most gratifying facts, and clearly show the advances made at the celebrated institution in which Mr. S. Tuke first commenced his philanthropic labours, and where the use of coercion may be now regarded as virtually abolished.

Other establishments might easily be quoted to prove the progressive advances recently made in the right direction; but we can only afford sufficient space at present for a few additional references, previous to giving our own opinion on the subject of restraint, which may still be considered, in some degree, although less so than recently, the *quæstio vexata* of the psychological branch of practical medicine. In Ireland, according to competent authority, the asylums are said to be as well, if not better, managed than many of those in England. At the Richmond Lunatic Asylum, in Dublin, for instance, according to the Irish Inspector-General's Report for 1843, personal restraint, as a part of the discipline of that hospital, had very much diminished for several previous years, and, as a general rule, was then done away with, although exceptions might occur. In the subsequent report for 1845, by the same public authorities, it is stated, that this asylum has fulfilled in every respect the humane and charitable intentions of the government; whilst at St. Patrick's Asylum, also in Dublin, the non-coercion system is pursued with success throughout the entire establishment. Again, at the Londonderry District Asylum, the non-coercion system is also practised successfully; and at the Maryborough Asylum, Dr. Jacob, in a recent report, announces that instrumental restraint has not been had recourse to, in a single instance, during the past year and a half; whilst several of the attendants have not even seen any apparatus for such a purpose.

From the lunatic asylums of Scotland, which are admirably conducted, and reflect much credit on the officers and superintendents, as well as honour upon the northern division of the United Kingdom, testimonies of a similar character might be easily produced. We, however, refrain from doing so, lest our remarks should, perhaps, appear to have extended to a greater length than some readers may approve. Nevertheless, Bethlem Hospital, the oldest establishment for the reception of lunatics in England, and whose history affords ample ground for comment; as, likewise, Hanwell Asylum, the most extensive institution of the kind in this country, and the locality where the practical application of total non-restraint is carried out most successfully, cannot be passed over in silence; we, therefore, make a few remarks upon each of these institutions.

To the condition of Bethlem Hospital at the early part of the present century we need not now refer, further than to state, that respecting the employment of restraint as an ordinary means of treating the inmates, nothing could well be worse, as demonstrated by the investigations of parliamentary committees. Since that period, matters have progressively improved, especially after the new hospital was erected; and still more so during recent years. In 1836, the number of patients at various periods under restraint in Bethlem Hospital amounted to 108, being nearly one-fourth of the whole number of lunatics confined; whilst the instances of restraint reported in the register amounted altogether to 495, the duration varying as much as the causes. In 1839, the average ratio of restraint fell to eleven patients per week, or 3·53 per cent. In 1842, the number became still more diminished, being three patients per week, or 0·81 per cent. In 1845, the proportion was only $1\frac{1}{3}$ patient per week, or 0·33 per cent.; whilst in 1848, the instances were so insignificant, that the rate fell to one-fifth—that is, about one patient in a month being under restraint; thus making the smallest ratio ever recorded in the annals of that institution, and proves incontrovertibly, that any opinions of an opposite description, which may elsewhere prevail, are erroneous. These facts are taken from the reports published by the authorities of Bethlem Hospital; and in support of a similar view, we would also refer to a communication made by Dr. Webster, in the number of the "Law Review," published last February, addressed to the committee on equity, in which it is stated that frequently not one patient among upwards of 400, treated at Bethlem Hospital, is under even temporary restraint of any kind. In the same document, Dr. Webster also remarks, in order to prove the evils of the former system, that in old Bethlem, when personal coercion was so common, and the mode of treating the insane patients was very different from the present method, suicides were, unfortunately, often met with. For instance, he states, during 20 years, ending the 31st December, 1770, out of 3629 patients admitted, 18 committed suicide, or one suicide in every 202 admissions. On the other hand, during 20 years, ending the 31st Dec., 1842, in 4676 admissions, only five suicides occurred, or one in every 925 insane patients. Again, during the first named period of 20 years, 55 patients "ran away" from the hospital, being one escape in every 66 patients admitted; whereas, during 20 years, ending the 31st December, 1842, only 16 patients escaped, or one in every 292 admissions, being one-fourth the previous amount; although the strait-waistcoat is now never employed, and the treatment of patients at new Bethlem is very different from what it was in the olden time; amusements, occupation, and much greater freedom, in addition to medical treatment, being often put in requisition to aid their recovery. The above statement forms a very agreeable contrast to the times when the unfortunate patient, Norris, with his iron collar, his double chain attaching him to the wall, and the trough, in which he lay on straw; as also to the miserable condition of almost every lunatic confined in the old institution, at Moor Fields. This was well depicted by the two celebrated statues of Caius Gabriel Cibber, which formerly figured upon the outer gate of the old hospital, but are now placed in the entrance hall of the new building in St. George's Fields; with the view, most likely, of showing how poor frail humanity was tortured in a former generation, compared with the treatment insane patients happily experience in these days of advanced civilization.

Unlike the establishment just mentioned, Hanwell Asylum has few reminiscences over which the authorities need wish to draw the veil of oblivion. Being a new institution, its annals are, therefore, of recent date; and as the modern system—thanks to the energy and zeal of Dr. Connolly—was there soon introduced, and fully carried into practical operation; the story is soon told respecting its efficacy in benefiting the patients, and the great influence its success exerted upon other asylums, and the medical profession, as, indeed, upon the whole community throughout the civilized world. Whatever doubts or fears previously prevailed upon the subject, in the minds of sceptical or timid individuals, have all now given way before well founded conviction, supported by ample experience. Upwards of eight years ago, Dr. Conolly decided upon making the apparently hazardous, but humane experiment of abolishing at once all kind of mechanical restraint from the Hanwell Asylum. The attempt was a bold one, but it has been eminently successful; and we are told, according to subsequent reports, up to the present time, that nothing has occurred to shake the confidence entertained respecting the efficacy or applicability of the system, amongst the authorities of that establishment, which now contains upwards of 1000 inmates, suffering under every form of mental disease.

Notwithstanding the experience thus acquired at Hanwell, the Commissioners in Lunacy considered it their duty to call attention, in their official Report of 1844, to the fact—by way of caution, doubtless, to the medical profession and the public—that since the autumn of 1842, two attendants had been killed, and three or four seriously injured, by dangerous patients, some of these injuries having happened in asylums where restraint was practised, and in others where non-restraint prevailed. In the same document, the Commissioners seem to approve of the system which admits occasional restraint, although they do not openly avow this to be their opinion. However qualified the notions then entertained at head quarters may have been respecting the important question of restraint, it is most satisfactory to find, by the last printed Report of the Commissioners in Lunacy, that they, like some authorities in other departments, march with the times, and announce progressive improvement. In the official document just quoted, the Commissioners state, that the instances of mechanical restraint in the public asylums are very few. Even in licensed houses, the practice of coercion is an exception to the general rule of treatment, which disavows it; and the modes of restraint now adopted are such as to pain and irritate the patient as little as is possible. The massive bars, the rings, and chains of iron, formerly resorted to, are no longer seen. Long continued coercion is not permitted; at the same time, the safeguards against lunatic patients being subjected to harsh or unnecessary restraint from the cruelty, idleness, or caprice of their attendants have been multiplied, and the chances of abuse reduced to a small amount. In short, the triumph of the non-restraint principle is almost accomplished, the number of opponents having dwindled down to insignificance; and in a few years more, as likely no combatants will be so bold as to take the field, or all parties being agreed, there must be an end of the controversy.

According to our views respecting the treatment of lunatics, the nearer we approach the principle of managing insane patients as if still rational beings, consistent with the safety of others and of themselves, so much the more likely will that system prove satisfactory. Kindness, conciliation, and the inspiring of confidence in the weakened mind of the poor sufferer towards the medical practitioner and attendants will frequently materially assist remedial measures. Three points we have always thought were of the utmost importance: 1st, never to deceive a lunatic, however insane; 2ndly, always to keep any promise when once made; of course being very cautious not to raise expectations which it would be wrong to gratify; and 3rdly, never to compromise the authority very properly inherent in the attending physician and superintendents.

Being decidedly in favour of the principle of non-restraint, we consider temporary seclusion in a darkened apartment, or the judicious confinement in a properly padded room, to be often of great benefit, when the patient is under violent excitement. But in such cases, the seclusion must only be had recourse to with the sanction of the medical officer; it should be carefully watched, its duration recorded, and the reasons distinctly stated why the restraint was employed. Some would, perhaps, consider seclusion in a padded room as a species of restraint, on similar grounds as the presence of one or more attendants in the same chamber with the maniac, to prevent his injuring himself and to calm his excitement, might be viewed as analogous; but the cases are not exactly parallel.

Padded rooms, made with strong waterproof ticking properly stuffed, or lined with Kamptulicon—a composition of cork and caoutchouc—ought to be constructed in every asylum for the insane, into which noisy, excited, or furious patients may be placed during a violent paroxysm. Experience has amply verified the utility of such apartments. At Hanwell, they are in frequent use when occasion requires, especially for epileptic patients; and we can speak from personal observation, there and elsewhere acquired, that rooms properly padded prove of great advantage in tranquillizing the patient in those kind of cases, which were formerly confined with hand-cuffs, ligatures, chains, and such like atrocities. Indeed, it is often remarkable how soon an excited maniac, or one who refuses food, will speedily become calm, or take nourishment, after being in temporary seclusion in properly padded rooms. One objection has been made to cells lined with Kamptulicon—viz., that in cold or frosty weather, the walls are sometimes not sufficiently elastic to obviate injury to the excited maniac, when he flings himself against them with violence, or knocks his head on the sides of the apartment. This inconvenience may, however, be very easily remedied by placing on the floor previously, either a chafing-dish with a hot iron plate; or, if the economy of the asylum permits, by introducing warm air into the room, a short time before admitting the patient. With this qualification, we consider padded cells thus constructed, or in the other mode, as indispensable, and they will be found most useful adjuncts to every asylum for the insane; indeed, none should be without similar apartments.

Numerous examples, illustrative of the highly injurious effects of personal coercion upon insane patients during the early stages of the attack, might be easily quoted. The experience of physicians conversant with the malady could easily furnish many instances of the kind, in which restraint was improperly employed; owing chiefly to the fears of relatives, or the erroneous notions entertained of what was proper; but oftener to the ignorance of attendants. From the cases recorded in the reports of public asylums, we only select two, as both are highly instructive, and show, in a marked manner, the effects of restraint. The first case is taken from the Bethlem Hospital Report for 1843; being that of a male patient brought for admission in a very violent and excited state, having, in addition to a strait-waistcoat, his arms bound with cords, his wrists secured by a belt, and his legs confined with strong webbing. In extenuation of such severe measures, a relative, who accompanied the lunatic, assured the steward that the restraint was absolutely necessary, "as he was very difficult to manage, and that it had even required as many as six men to place him under coercion." The first thing done on admission was to release the patient from all restraint; and although, as might be expected, he remained for some days in a highly excited state, so as to require the constant watching of one, and sometimes two attendants, no personal coercion was afterwards used during the whole time he remained under treatment. In a few days, symptoms of an inflammatory affection of the chest appeared, from the effects of which, combined with great cerebral excitement, he died, in a fortnight after admission. A post-mortem examination of the body proved that the breast-bone and one rib were fractured; the interior of the chest was also found much affected, in consequence of the irritation which the broken bones produced on the lining membrane, and it can hardly be doubted, that these severe injuries occurred in the struggles which took place when so much restraint was imposed.

The second case is copied from Dr. Conolly's Report to the Middlesex Magistrates for 1841, and is that of "a male patient admitted on the 23rd of June, when he was reported to be dangerous to those about him. It was averred, that in the asylum from whence he came, he had been kept almost constantly in instrumental restraint for three months. He laboured under some religious and other delusions, was almost always talking, and somewhat restless in his habits, and it was not practical to keep him among the quiet and feeble patients. He was placed, on that account, in a ward assigned to more troublesome patients, but he was, of course, never subjected to restraint; and although fidgety, and always in action, he occasioned so little solicitude to attendants trained to habitual vigilance, that he was never once even put in seclusion." In September, this patient begun to work in the garden, was placed in one of the quietest wards, wrote affectionate and rational letters to his wife; and latterly, he partook of the sacrament in the chapel, at his own request. The instances now recorded, are only samples of many others of a similar description, which we could easily detail, but forbear, lest our readers' patience should be exhausted with what might perhaps appear supererogation.

But we must now take leave of the whole subject, however easy it would be to enlarge upon the many interesting and important topics, which have now passed under review. Devoid of all prejudice, desirous of acquiring information from other observers, yet most anxious for the diffusion of sound practical knowledge, we have thrown together, in the previous pages, those general conclusions which we have adopted after considerable, if not careful reading of various authors, verified by our own experience, and also from personally observing the practice pursued by different practitioners in British as well as in continental asylums for the insane. Supported by many convincing proofs of the injurious effects of the old system, sustained by the daily accumulating evidence of experienced and truth-seeking physicians against its employment, and encouraged by the numerous conversions from the ranks of active opponents of non-restraint into that of advocates of the new doctrine, our convictions are confirmed. Seeing likewise on almost every side, the great benefits which have been conferred upon afflicted lunatics, by adopting the more humane and non-coercive plan of management, aided by moral means and appropriate medical treatment, we hesitate not a moment to proclaim, amongst the many philanthropic movements so characteristic of the present age, none stands more prominently forward, or deserves higher praise, than the modern system of treating those fellow creatures who have unfortunately become attacked by the saddest of all diseases—mental aberration. The cure, or even the alleviation of this truly calamitous malady, which often reduces the sufferer, for the time, to an apparently lower scale in the animal creation, although hitherto endowed with the highest intellectual attributes, and inspired with life and feeling by his Divine Maker, is indeed real charity, notwithstanding it may be only possible to mitigate the patient's severe afflictions. Honour and thanks are therefore most justly due to the illustrious and humane combatants in the glorious cause of suffering humanity, who have bravely fought the great fight, have persevered through bad report or good report, have never flinched from asserting the high principle for which they pledged their faith, risked worldly fortune, and perilled their future fame.

